# A computational fluid–structure interaction model to predict the biomechanical properties of the artificial functionally graded aorta

**DOI:** 10.1042/BSR20160468

**Published:** 2016-12-23

**Authors:** Arezoo Khosravi, Milad Salimi Bani, Hossein Bahreinizade, Alireza Karimi

**Affiliations:** * Atherosclerosis Research Center, Baqiyatallah University of Medical Science, Tehran 15897, Iran; † School of Mechanical Engineering, Iran University of Science and Technology, Tehran 16887, Iran; ‡ Department of Mechanical Engineering, Sahand University of Technology, Tabriz, Iran; § Basir Eye Health Research Center, Tehran 14186, Iran

**Keywords:** artificial vessel, finite element method, functionally graded materials, thick cylinder

## Abstract

In the present study, three layers of the ascending aorta in respect to the time and space at various blood pressures have been simulated. Two well-known commercial finite element (FE) software have used to be able to provide a range of reliable numerical results while independent on the software type. The radial displacement compared with the time as well as the peripheral stress and von Mises stress of the aorta have calculated. The aorta model was validated using the differential quadrature method (DQM) solution and, then, in order to design functionally graded materials (FGMs) with different heterogeneous indexes for the artificial vessel, two different materials have been employed. Fluid–structure interaction (FSI) simulation has been carried out on the FGM and a natural vessel of the human body. The heterogeneous index defines the variation of the length in a function. The blood pressure was considered to be a function of both the time and location. Finally, the response characteristics of functionally graded biomaterials (FGBMs) models with different values of heterogeneous material parameters were determined and compared with the behaviour of a natural vessel. The results showed a very good agreement between the numerical findings of the FGM materials and that of the natural vessel. The findings of the present study may have implications not only to understand the performance of different FGMs in bearing the stress and deformation in comparison with the natural human vessels, but also to provide information for the biomaterials expert to be able to select a suitable material as an implant for the aorta.

## INTRODUCTION

Artificial vessels can be considered as the replace parts of natural vessels as a result of vascular diseases, such as the aneurysm or atherosclerosis. Grafts of artificial vessels are the common treatment methods with high publicity in the world. Considering the geometry and structure of the natural vessels, a thick wall cylinder can be a suitable estimation of a blood vessel for the finite element (FE) simulation purposes [1,2]. However, accurate 3D models of the blood vessels have also being used for the blood vessel simulation [3–6].

In the recent years, the application of functionally graded materials (FGMs) in bioengineering has grown so that different models have been proposed for these materials. Specifically in this application, use of composite materials has certain problems like inability to satisfy solid and fluid conditions simultaneously, stress magnitudes far from natural values, the separation between the layers and sudden changes of mechanical properties [7,8]. Considering these problems, replacement of conventional materials in artificial vessels with smart biomaterials has been studied. Main reason for using the FGM is to satisfy difficult and somehow antithetical conditions. A smart and continuous combination of different biomaterials with different mechanical, thermal and magnetic properties can satisfy design needs and also prevent undesired events like stress concentration in the structures. In the early 21st century, a significant amount of analytical studies have been performed on various applications of FGMs, including the simulation of biomechanical functionally graded biomaterial (FGBM) structures. For instance, FGBMs have been used in dental implants [9], and replacement of damaged parts in various parts of the body caused by trauma or other disorders, such as intervertebral disc degeneration [10], prosthetic knees [11] or holes of hip acetabular bone anchor [12].

Considering the aorta's geometry, cylindrical FGBM was used to simulate a natural vessel. Lame, for the first time in 1852, presented the exact solution of axial axisymmetric thick-wall cylinders with homogeneous and isotropic properties under uniform pressure. They found the stress and displacement distributions of a hollow cylinder via the plane elasticity theory [13]. Fukui and Yamanaka [14] in 1992 derived governing equations on the functionally graded thick-walled tubes under an internal pressure from the Lame equations and found a numerical solution for that. Horgan and Chan [15] in 1999 extracted the equivalent of a hollow FGM cylinder in a state of plane strain in the radial direction and determined the stress distribution. Tutuncu and Ozturk [16] in 2001 represented the exact solution of fixed-wall cylindrical and spherical vessel in response to the pressure. Jabbari et al. [17,18] investigated the mechanical and thermal stresses of a hollow cylinder under symmetrical and asymmetrical loadings. Gao et al. [19] also analysed the stress on the aortic arch in an actual vessel model under pulsatile blood flow. Xiang et al. [20] surveyed the exact solution of a hollow cylinder with radially linear variations of mechanical properties. Moreover, they studied FGM cylinder by assuming multi-layered one and compared with Tutuncu solution [21]. Tutuncu [22] by considering the exponential variation of the modulus of elasticity, calculated the stress distributions in an inhomogeneous cylindrical structure. Setoodeh et al. [23] solved an axisymmetric cylindrical shell under time-varying stress with the differential quadrature method (DQM) and the results were compared with the FE outcomes. In the same year, they have also solved free vibration of axisymmetric cylindrical shell under time-varying thickness with DQM and the results were compared with the FE ones [24]. Khoshgoftar et al. [25] solved the general equations of tick-wall FGM cylinder under different pressures using FSDT method and compared them with the obtained results of plane elasticity.

In the present study, the biomechanical behaviours of real and artificial FGBM vessel under time-varying pressure were investigated and, finally, the obtained results were compared and discussed.

## MATERIALS AND METHODS

**Figure 1:**
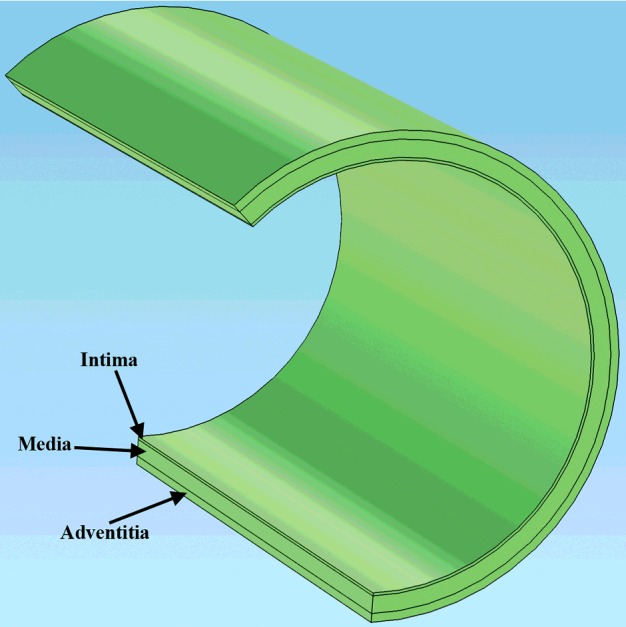
The FE model of the blood vessel, named as thick cylinder

An aorta vessel with elastic FGM walls was simulated and its mechanical behaviour was investigated in FE software. The results of this simulation have used to suggest a proper design for an artificial blood vessel to replace parts of aorta. According to the vivid results in the literature of elastic vessel, the used simulator code obtained from DQM was validated with FEM results. Thick wall geometrical model of the aorta is shown in Figure 1. Some specifications of the aorta's geometry and its mechanical properties are listed in Table 1. Equations (1)–(4) are respectively displaying the variation of the density, alteration of the elastic modulus and dimensionless stresses equations [23]. In these equations, *T* is the loading time, *h* is the thickness and *L* is the length of vessel.

1 $$\rho \left(r\right)=8900{\left(\frac{r}{0.08}\right)}^{-5.93}$$

2 $$E\left(r\right)=223\times 10^{9}{\left(\frac{r}{0.08}\right)}^{2}$$

3 $$P\left(t\right)=P_{0}\sin\left(\pi t\right)$$

4 $$\begin{array}{c} T=\frac{t}{t_{e}},Z=\frac{z}{L},R=\frac{r}{r_{i}},U_{r}=\frac{u_{r}\times K}{P_{0}\times h},K=10\;\;GPa\\ S_{i}=\frac{\sigma_{i}}{P_{0}}\left(i=r,z,t\right) \end{array}$$

**Table 1:** Mechanical properties of the elastic vessel's wall [23]

	Poisson's ratio	Internal radius (m)	External radius (m)
Value	0.3	0.8	1.0

In the next step, a mesh was generated on the geometry and then the boundary conditions were applied on the model. The pressure was considered to be time dependent.

**Figure 2:**
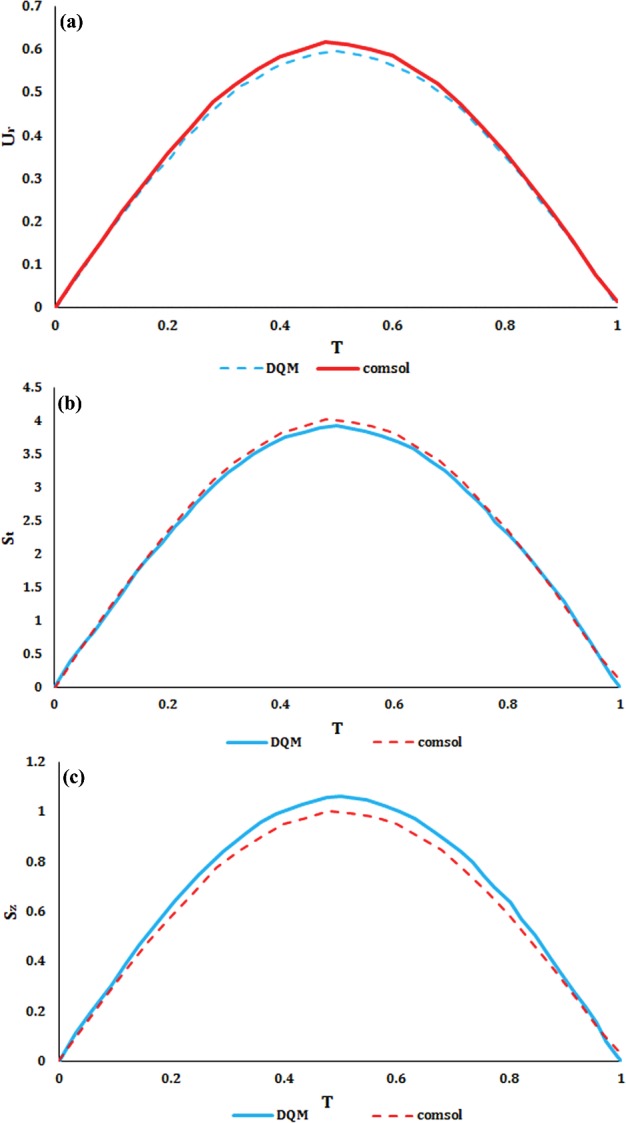
The FE results of the (**a**) radial displacement, (**b**) axial stress and (**c**) circumferential stress of an elastic vessel compared with the DQM method [23]

Comparison between DQM and numerical results has been shown in Figure 2. Numerical results error in the radial displacement was 3.017%, in the peripheral stress was 1.962% and in the longitudinal stress was 5.99%. Therefore, the errors of FEM were rather low and these results can be used for further analysis. In the other words, FE simulation with described specifications can be used to analyse the mechanical behaviour of the aorta vessel.

Equations (5)–(8) are respectively Hooke's law, Poiseuille's fluid flow equation and stress and strain equations. In these equations, *Q* is the flow rate and μ is the dynamic viscosity. Equations (9)–(11) can be derived from eqns (5)–(8).

5 σ=Eɛ

6 $$\frac{dP}{dx}=-\frac{8\mu Q}{\pi r_{x}^{4}}$$

7 $$\sigma =\frac{P_{\left(x\right)}r_{\left(x\right)}}{t}$$

8 $$ɛ=\frac{r_{\left(x\right)}-r_{\mathrm{in}}}{r_{\mathrm{in}}}$$

9 $$P=\left(P_{0\left(t\right)}-\frac{E_{\mathrm{in}}t}{r_{\mathrm{out}}}\right)e^{-\frac{2\tau x}{E_{\mathrm{in}}t}}+\frac{E_{\mathrm{in}}t}{r_{\mathrm{out}}}$$

10 $$\tau =\mu \frac{U}{d}$$

11 $$\frac{U}{d}=Q$$

**Figure 3:**
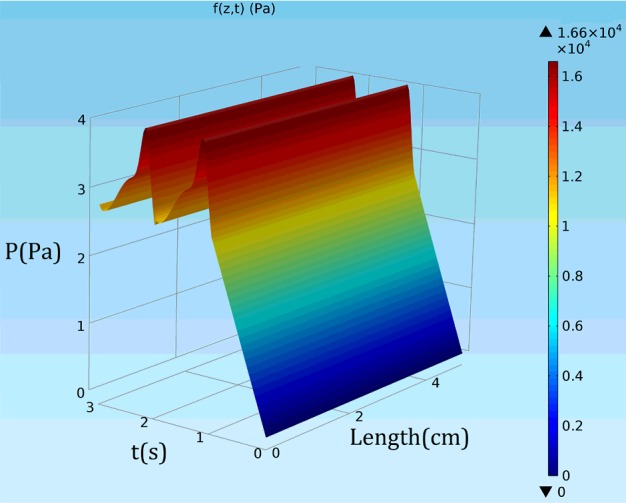
The variation of the pressure compared with the time (s) and length (m)

**Figure 4:**
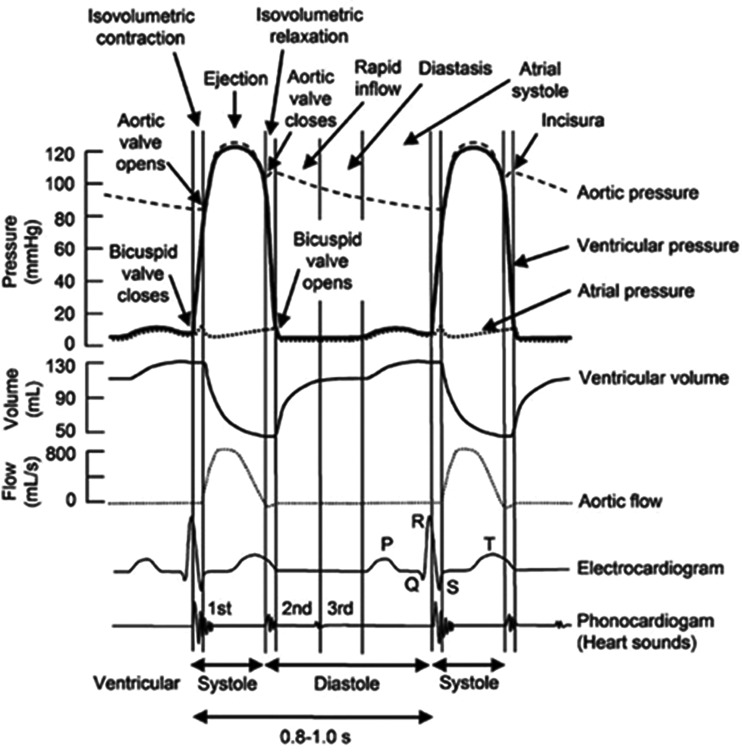
The variation of the pressure in a heart cycle [37]

**Figure 5:**
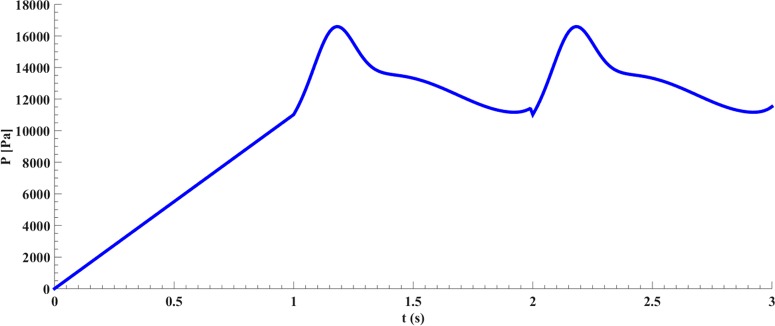
The inlet pressure of a natural aorta compared with time [37]

**Figure 6:**
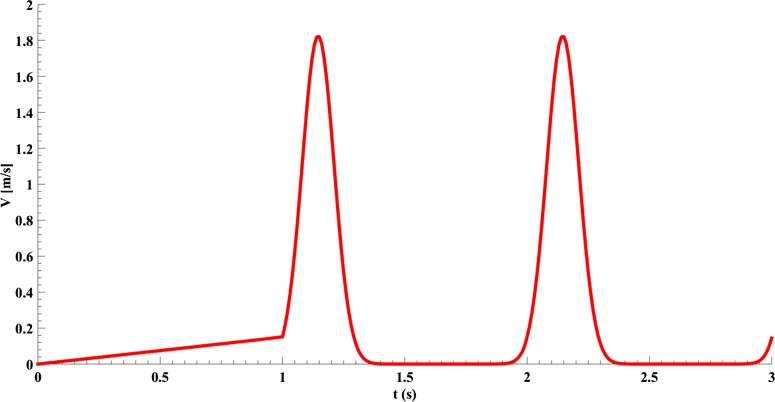
The inlet velocity of a natural aorta compared with time [37]

In eqns (9)–(11), *P* is time dependent pressure in the inlet, *E*_in_ is elastic modulus in inner radius, *t* is thickness, *r*_in_ is the internal radius, τ is the shear stress, *Q* is the ratio of velocity to passed distance by the flow in the wall and μ is the blood's dynamic viscosity. Magnitudes of these parameters are provided in Table 2. The variation of the pressure compared with the time (s) and length (m) is presented in Figure 3. In addition, the variation of the pressure in a heart cycle is depicted in Figure 4. The initial pressure was determined and plotted against the time according to Figure 5. In addition, the variation in the velocity compared with the time is also plotted in Figure 6.

**Table 2:** Mechanical properties of blood flow [19]

	*P*_0(_*_t_*_)_ (Pa)	*t* (mm)	*Q* (s^−1^)	μ (Pa·s)
Value	16000	2.32	11.91	0.0035

In order to determine the accuracy of the model with time dependent pressure, this model was solved as fluid–structure interaction (FSI) using commercial FE software ADINA. In this model, the flow considered to be incompressible and laminar. Furthermore, the blood flow was considered to be unsteady [26]. In addition, due to a large diameter of aorta, blood was considered to be Newtonian.

In this model, there was a downward gravity force (9.81 m/s^2^) [27,28]. Eight hundred and eighty elements were applied on the model in the length and thickness directions respectively, which were types of 2D solid and rule-based with four points in each element. No slip boundary condition was used for walls of aorta [29,30]. This mesh generation have an acceptable error of 3% [31,32]. The number of elements in solid and fluid phases were determined to reduce the difference between average velocity of fluid and blood vessel's wall displacement below 0.001 [33,34]. Mechanical properties of the blood are summarized in Table 3. Moreover, the simulation was two-way coupled [35,36].

**Table 3:** Some of the aorta vessel's properties [19]

	Poisson's ratio	Density ( 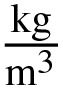 )	Internal radius (m)	External radius (m)	Length (m)
Value	0.5	1090	0.0168	0.01912	0.05

**Figure 7:**
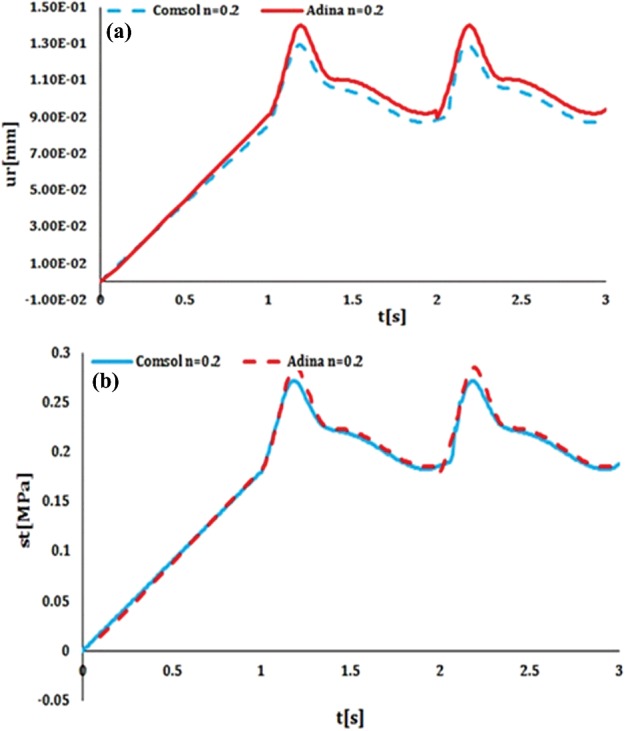
Comparison of the (**a**) radial displacement and (**b**) circumferential stress of an elastic vessel on the basis of FSI simulation These results are reported in the middle of the vessel's thickness and the length of vessel at the time of 2.2 s. The heterogeneous index of 0.2 was used for both models.

Results obtained from the FSI model with time dependent pressure, solved in COMSOL and ADINA, and were compared in order to validate the model. Comparison of the radial displacement and circumferential stress between COMSOL and ADINA results has been shown in Figure 7. Maximum error for the circumferential stress compared with the time was 8.93% and for the radial displacement compared with vessel's length (in 2–3 s) was 4.68%. Therefore, FE simulator can accurately model fluid flow with time dependent pressure.

Main goal of the present study was to investigate the displacement and stress change with heterogeneous index and find a proper heterogeneous index. FGBM vessels were considered to vary radially and gradually from Dacron to polyurethane based on continuously nonlinear parabolic forms.

Natural vessel's model was considered to be consisted of three layers with respective thickness of 1, 6 and 3 for the intima, media and adventitia respectively. Mechanical properties of these layers are illustrated in Figure 9 and Table 3 [19].

In all of the simulations performed in the present study for the time and location dependent pressure, inlet and outlet of the blood vessel considered to be fixed. Mesh was generated as mapped mesh and the simulation was time dependent and two-way coupled. In order to reach to mesh independency, mesh size of 0.15 mm with relative error of 2.99% was used. Results of mesh independency test are shown in Table 4. Mechanical properties of material used in FGMs are also listed in Table 5.

**Table 4:** Mesh independency results for von Mises stress

Mesh size (mm)	0.2	0.15	0.1
Relative error	4.93	2.76	2.57

**Table 5:** Mechanical properties of materials used in the FGM [38]

	PU	Dacron
*E* (MPa) [2]	25	1.9
ν	0.49	0.37
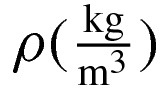	1200	1380

## RESULTS AND DISCUSSIONS

**Figure 8:**
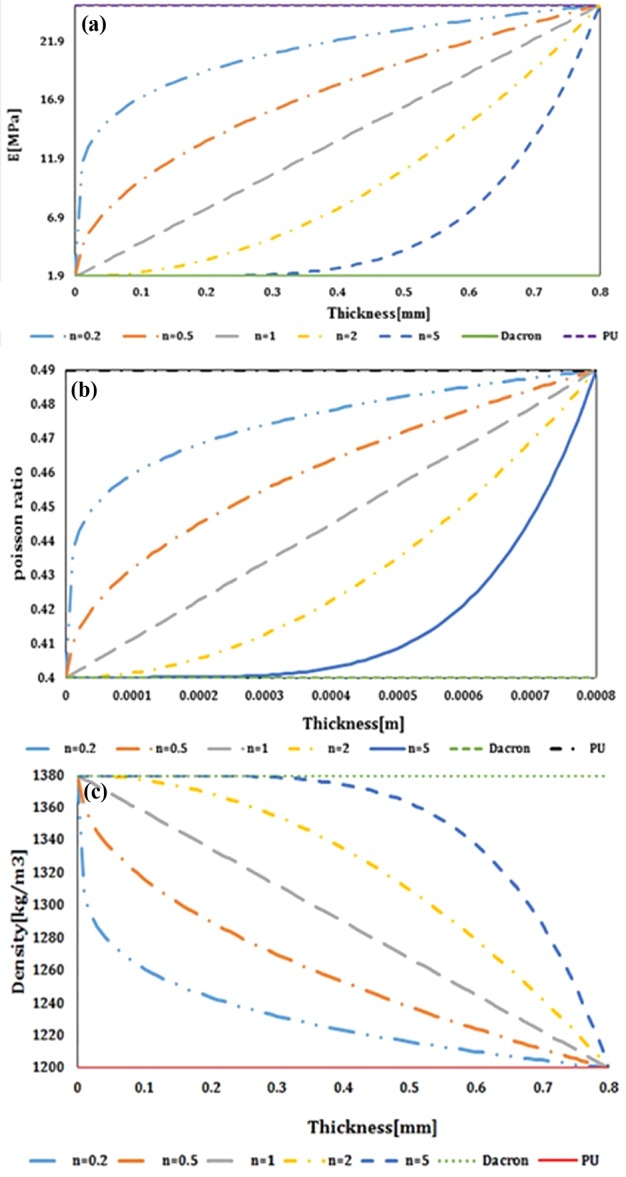
The variation of the (**a**) elastic modulus, (**b**) Poisson's ratio and (**c**) density compared with the heterogeneous index in parabolic FGM

**Figure 9:**
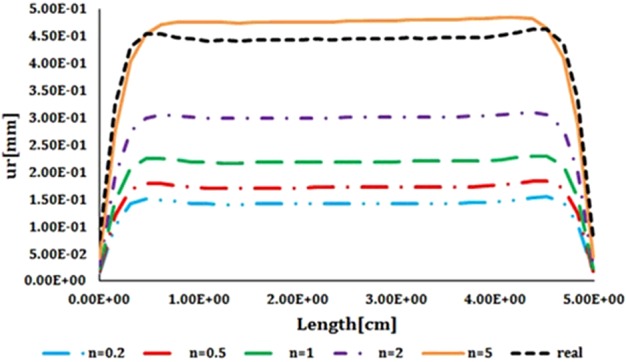
Comparison of the radial displacement compared with the length in FGM artificial vessel with different heterogeneous indexes and natural vessel's behaviour

As the FSI simulation in ADINA software was validated with analytical results, these results can be considered to be reliable. The variation of the elastic modulus, Poisson's ratio and density compared with the thickness of the thick vessel are presented in Figure 8. The results revealed that a lower heterogeneous index invokes a higher elastic modulus as well as the Poisson's ratio at the initial zone of the diagrams. However, at the end of the diagram all curves reach together and, as a result, the same outcomes were observed. Regarding the variance of the density in respect to the thickness, it is revealed that a lower heterogeneous index would have a higher density whereas by increasing the thickness this value is decreasing accordingly. The radial displacement compared with the length of the thick vessel is exhibited in Figure 9. The results showed an increasing, constant and decreasing behaviour for the radial displacement by continuing during the length of the vessel regardless of the heterogeneous index.

**Figure 10:**
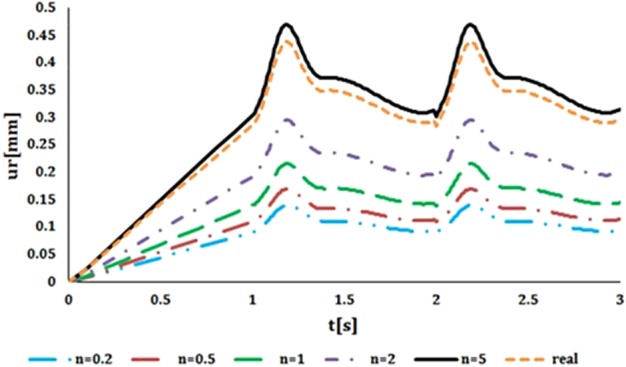
Comparison of the radial displacement compared with the time in FGM artificial vessel with different heterogeneous indexes and natural vessel's behaviour

**Figure 11:**
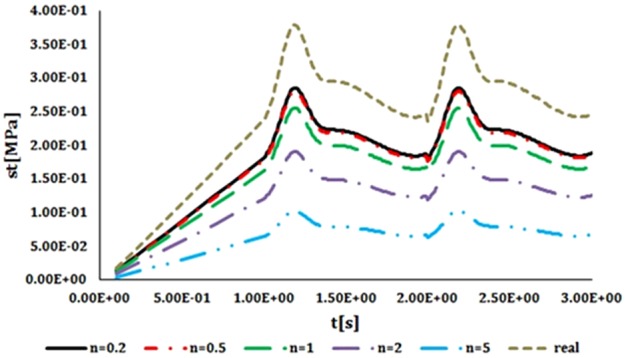
Comparison of the circumferential stress compared with the time in FGM artificial vessel with different heterogeneous indexes and natural vessel's behaviour

For both radial displacements compared with the time, closest displacement to natural vessel was resulted from heterogeneous index of 5 (Figure 10). As heterogeneous index lowers difference between artificial and natural vessel's displacements becomes larger. Results of circumferential stress suggest the opposite (Figure 11). Heterogeneous index of below 0.2 shows the closest circumferential stress to the natural vessel. But, even in this case, FGM artificial vessel with heterogeneous index of 5 and higher have rather low stress. Even though these values are significantly different from natural vessel but should not cause any problem.

**Figure 12:**
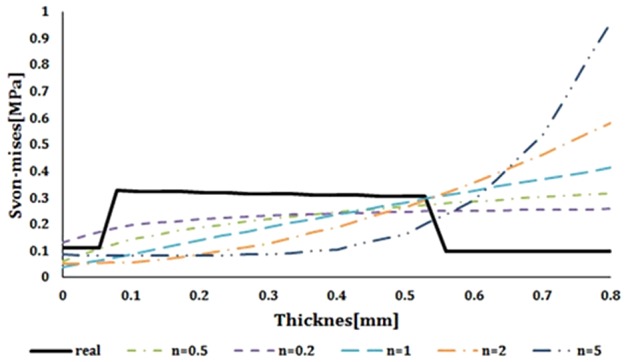
Comparison of the von Mises stress compared with the thickness in FGM artificial vessel with different heterogeneous indexes and natural vessel's behaviour

Results of the von Mises stress compared with thickness for natural vessel and FGM artificial vessels with different heterogeneous indexes have been shown in Figure 12. Results of the present study suggest that lower heterogeneous indexes can results in closer von Mises stress to natural vessel. But this can lead to stress shielding which is an undesired effect. Furthermore, because of three-layer nature of natural vessel, there is a sudden change in the mechanical behaviour between these layers. It should be noticed that this sudden change will occur in all stresses and displacements. This effect is the main problem with using composite materials for the artificial vessel.

**Figure 13:**
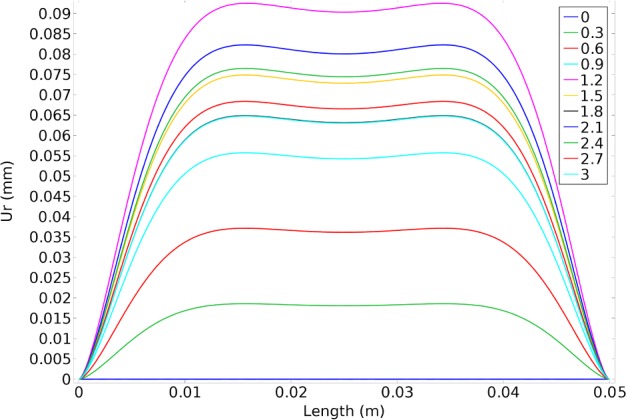
The radial displacement compared with the length at different times

**Figure 14:**
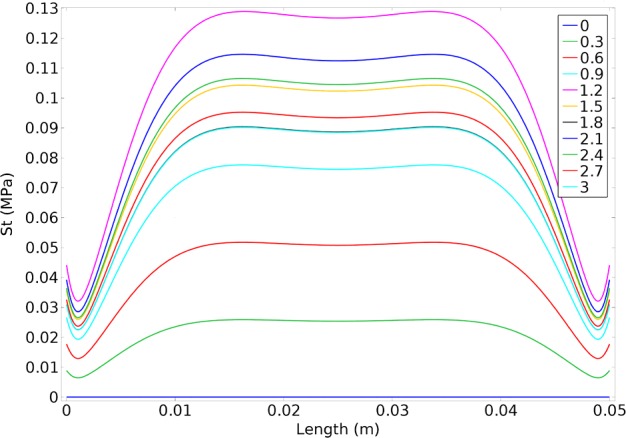
The circumferential stress compared with the length in different times

In conclusion, if the aim of the design is to reach a better stress then, FGM with lower heterogeneous indexes would be preferable. But if the goal of the design is to reach a better displacement then, higher heterogeneous indexes would be better. If both conditions of stress and displacement should be met, then results of the present study suggest an intermediate heterogeneous index for artificial vessel. Results of radial displacement and circumferential stress compared with thickness in different times and results from the COMSOL simulation are displayed in Figures 13 and 14 which will approve our conclusion. Maximum values of both radial displacement and circumferential stress happened at 1.2 s and 2.2 s respectively.

## CONCLUSION

In the present study, the mechanical behaviour of parabolic FGM artificial vessel was compared with the natural blood vessel mechanical behaviour. Blood considering to be both time and location dependent. It should be noticed that, manufacturing a composite artificial vessel like the natural blood vessel is not possible due to unavailability of materials with mechanical properties similar to that of three layers of natural vessel. Therefore, the aim of the present study was to find a suitable substitute using FGMs for artificial vessel. In addition, radial displacement, circumferential stress and von Mises stress were selected as behaviour characteristics and were attempted to minimize these parameters of artificial vessel be close to natural vessel. By comparison of the mechanical behaviour of the model based on the homogeneous materials and models based on FGMs, models based on FGMs showed closer performance to the natural vessel. As a result of increasing the heterogeneous index, some of behavioural characteristics were improved and some were worsened, results of this suggest that an intermediate heterogeneous index would be best for most design purposes.

## Abbreviations

DQM: differential quadrature method
FE: finite element
FGBM: functionally graded biomaterial
FGM: functionally graded material
FSI: fluid–structure interaction
